# Altered consciousness mediates the effect of hypernatremia, but not hyponatremia, on mortality among patients observed in the ICU

**DOI:** 10.21203/rs.3.rs-3255987/v1

**Published:** 2023-08-18

**Authors:** Miguel Ángel Armengol de la Hoz, Sicheng Hao, Nathan Hutzel Raines, Leo Anthony Celi, Patricia Sánchez-González, Enrique J. Gómez, John Danziger

**Affiliations:** 1. Big Data Department, PMC, Progress and Health Foundation (FPS), Regional Ministry of Health of Andalucia; 2. MIT Critical Data, Laboratory for Computational Physiology, Institute for Medical Engineering and Science, Massachusetts Institute of Technology, Cambridge, Massachusetts.; 3. Department of Medicine, Beth Israel Deaconess Medical Center, Boston, Massachusetts, United States; 4. Biomedical Engineering and Telemedicine Centre, Center for Biomedical Technology, ETSI Telecomunicación, Universidad Politécnica de Madrid, Madrid, Spain.; 5. Centro de Investigación Biomédica en Red de Bioingeniería, Biomateriales y Nanomedicina, Instituto de Salud Carlos III, Madrid, Spain

**Keywords:** Dysnatremia, Hyponatremia, Hypernatremia, eICU, Glasgow Coma Scale, mortality

## Abstract

**Purpose:**

Dysnatremias - hypernatremia and hyponatremia - may be associated with mortality through their impact on altered consciousness. We examined the mediating effect of decreased consciousness on the relationship between dysnatremia and mortality.

**Methods:**

Among 195,568 critically ill patients in the United States contained in the eICU database, we categorized serum sodium into bands of 5mEq/L. Using causal mediation analysis, we compared bands in the hypernatremia and hyponatremia ranges to a reference band of 135–139mEq/L to determine the proportion of mortality mediated by decreased consciousness as determined by the Glasgow Coma Score (GCS).

**Results:**

Both hyponatremia (OR [95%CI] for bands: <120mEq/L: 1.58 [1.26–1.97]; 120-<125mEq/L: 1.92 [1.64–2.25]; 125-<130mEq/L: 1.76 [1.60–1.93]; 130-<135mEq/L: 1.32 [1.24–1.41]) and hypernatremia (OR [95%CI] for bands: 140-<145mEq/L: 1.12 [1.05–1.19]; 145-<150mEq/L: 1.89 [1.70–2.11]; ≥150mEq/L: 1.86 [1.57–2.19]) were significantly associated with increased mortality. GCS mediated the effect of hypernatremia on mortality risk (Proportion mediated [95%CI]: 140–144mEq/L: 0.38 [0.23 to 0.89]; 145–149mEq/L: 0.27 [0.22 to 0.34]; ≥150mEq/L: 0.53 [0.41 to 0.81]) but not hyponatremia (proportion mediated 95%CI upper bound <0.05 for all bands).

**Conclusion:**

Decreased consciousness mediates the association between increased mortality and hypernatremia, but not hyponatremia. Further studies are needed to explore neurologic mechanisms and directionality in this relationship.

## Introduction

Water homeostasis in humans is tightly regulated by a cascade of neurohormonal and renal mechanisms. Disorders of water imbalance, characterized clinically as dysnatremias, result in alterations of cell size and function [1]. Both hyponatremia and hypernatremia have been linked to a range of neurologic sequelae, including cognitive dysfunction [2, 3], physical impairment [4] and dementia [5]. These sequelae are thought to arise as a consequence of swelling and shrinkage of cells in the central nervous system and are associated with increased mortality [6–9]. Restoration of water balance to normalize osmotic forces acting on these cells is one of the cardinal therapies in treating dysnatremia [10].

At present, it remains unclear whether the association between dysnatremia and mortality is driven by consequences of the dysnatremia itself, or driven by the underlying disease that leads to the water imbalance [11–13]. Given that water retaining pathophysiologic mechanisms, such as sensed volume mediated stimulation of vasopressin, are complex and often challenging to measure, it can be difficult to account for confounding due to illness severity in investigating the association between dysnatremia and death.

To better understand the relationship between dysnatremia and mortality, we tested what proportion of the association between dysnatremia and increased mortality was mediated by change in level of consciousness after controlling for differences in underlying illness severity. We hypothesized that, through changes in neuronal size and function caused by dysnatremia which were not directly measured in this study but which would manifest as an abnormal Glasgow Coma Score (GCS) [14], decreased consciousness would mediate the association between mortality and both hypernatremia and hyponatremia.

## Methods

This is a retrospective cohort study of adult patients admitted to intensive care units (ICUs) in the United States. Eligible participants were individuals included in the “eICU Collaborative Research Database” (eICU) who had data on serum sodium and GCS during their first day of ICU admission; had a known disposition on hospital discharge; and had not previously been in the ICU during their hospital admission. Inclusion criteria for the eICU database overall have been previously described [15–17].

### Data Source

Philips Healthcare, a major vendor of ICU equipment and services, provides a telehealth ICU platform to over 300 hospitals across the United States. Data from participating hospitals is anonymously curated in the eICU Research Institute database, a collaborative partnership between Philips Healthcare and the Laboratory for Computational Physiology at Massachusetts Institute of Technology [15–17]. It contains high-resolution patient data including demographics, vital signs, laboratory tests, illness severity scores, fluid intake and outputs, and diagnostic coding from patients admitted between 2003 and 2016.

The data in eICU has been previously de-identified, and the institutional review boards of the Massachusetts Institute of Technology (No. 0403000206) and Beth Israel Deaconess Medical Center (2001-P-001699/14) both approved the use of the database for research. Re-identification risk has been certified as meeting safe harbor standards by an independent privacy expert (Privacert, Cambridge, MA) (Health Insurance Portability and Accountability Act Certification no. 1031219–2).

### Exposure

The primary exposure was the first serum sodium concentration measured following admission to the ICU, categorized into eight different five mEq/L increments: <120, 120-<125, 125-<130, 130-<135, 135-<140, 140-<145, 145-<150, and ≥150. The 135-<140mEq/L group was considered the reference group, with sodium concentrations higher than the reference group considered “hypernatremic” and concentrations lower than the reference group considered “hyponatremic.” The mediating exposure of interest was the lowest GCS score obtained within 24 hours of admission to the ICU (“GCS baseline”).

### Outcome

The primary outcome of interest was in-hospital mortality during the entire index hospitalization.

### Variables Included in Analyses

In addition to serum sodium category, out model incorporated the demographic variables age, gender, and race coded as caucasian or not caucasian; underlying illness severity as measured by the Apache II scoring system [18], with serum sodium and GCS removed from the score to minimize collinearity with our exposure and outcome variables of interest; and admission diagnosis or diagnoses as adjudicated by trained clinicians within the first 24 hours of ICU admission. Admission diagnosis was further categorized into the ten most common clinical categories: sepsis, cardiac arrest, cerebrovascular accident, acute coronary syndrome, gastrointestinal bleed, congestive heart failure, trauma, pneumonia, other respiratory disease, and other non-respiratory disease. Because of their relationship with dysnatremia, a history of cirrhosis or congestive heart failure as defined by Charlson comorbidity index criteria [19] were also included in the analysis as separate variables. ICU type (medical, medical/surgical, surgical, cardiac, cardiothoracic, and neurological) was included in the model as a categorical variable. Mechanical ventilation during the first 24 hours of care was included as a binary variable.

We used median imputation for continuous variables with missing values. This technique preserves the central tendency of the data and minimizes the impact of potential outliers. Missing values for binary variables were replaced with “0”.

### Statistical Analyses

**Fig 1:**
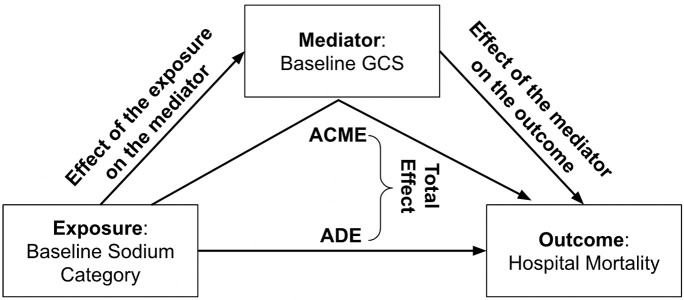
Graphical representation of our mediation analysis, with a potential mechanism linking hyponatremia with increased mortality through altered mental status. Exposure is baseline sodium, which we divided into 8 categories with 135 to 139 mEq/L as the reference level. The mediator of interest is baseline Glasgow Coma Scale (GCS) score, a measure of altered mental status. The outcome is hospital mortality. Average causal mediation effect (ACME) is the indirect effect of baseline sodium category on hospital mortality mediated by the effect of GCS score. Average direct effect (ADE) is the direct effect of baseline sodium category on mortality independent of its effect mediated by GCS. Total effect is the sum of ADE and ACME. All models were adjusted for age, gender, ethnicity, illness severity, ICU Type, admission diagnosis, history of cirrhosis or congestive heart failure, and mechanical ventilation during the first 24 hours of care

We performed a causal mediation analysis in order to determine to what degree the association between serum sodium group and hospital mortality in our cohort was mediated by GCS (Figure 1), controlling for the covariates discussed above [20, 21]. In this analysis, the “total effect” represents the overall association between sodium level and mortality. The “average causal mediation effect” (ACME) represents the indirect effect of sodium grouping on mortality which is *dependent on the effect of sodium group on GCS (the “mediator”) and the effect of GCS on mortality*. The “average direct effect” (ADE) represents the direct effect of sodium grouping on mortality *independent from the GCS score*, and can be calculated by subtracting the ACME from the total effect. The “proportion mediated” statistic is the proportion of the total effect explained by the mediator, calculated as ACME divided by total effect. For the mediation analysis, we used linear regression to estimate the association between sodium group and GCS and logistic regression to estimate the effect of both serum sodium and GCS on hospital mortality.

We used the “mediation” package in R to perform the causal mediation analysis [22]. 95% confidence intervals of total effect, direct and indirect effect were estimated using the Bootstrap method with 1,000 repetitions. We assumed sequential ignorability conditions [21] were satisfied and performed sensitivity analyses to confirm this assumption’s robustness. Statistical significance was determined using P.<..05. Analyses were conducted using R version 3.6 (R Project for Statistical Computing).

## Results

**Fig 2:**
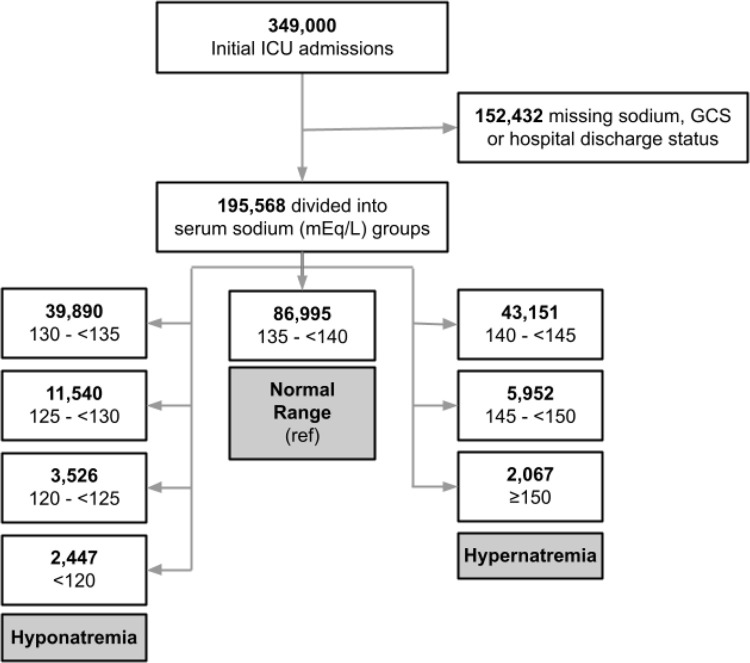
Participant flowsheet. Sodium groups are divided based on serum sodium in mEq/L. Abbreviations: ICU, intensive care unit; GCS, Glasgow Coma Scale

Participant selection and sodium category assignment is shown in Figure 2. After exclusions, among 195,568 critically ill patients in ICUs across the United States, 57,403 (29.4%) were hyponatremic and 51,170 (26.2%) were hypernatremic. Overall mortality was 9.4% (n = 18,469). Participant characteristics stratified by sodium category are presented in Table 1, with additional parameters included in the models shown in **Supplemental Table 1**.

Individuals with severe hypernatremia (serum Na ≥ 150mEq/L) had sepsis as their admission diagnosis more frequently than other groups; had cardiovascular-related admission diagnoses less frequently; were marginally older on average; and were more frequently non-caucasian. Individuals in all 3 hypernatremia groups were intubated at the time of admission to the ICU more frequently than individuals in other groups. Individuals in the hyponatremia groups had a diagnosis of cirrhosis more frequently than individuals in other groups.

**Fig 3:**
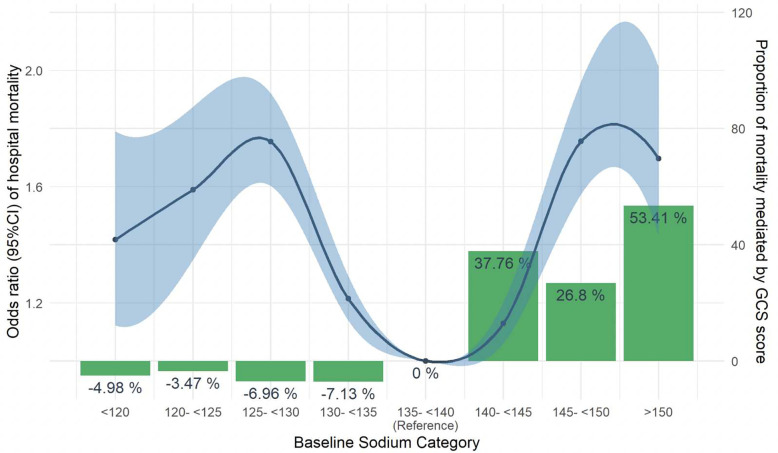
Mediating effect of decreased consciousness on the association between sodium concentrations and mortality. The blue line with ribbon represents the odds ratio and 95%CI for hospital mortality for each sodium group compared to the 135–<140mEq/L reference group, and shows increased odds for mortality across all dysnatremia groups. The green bars represent the percent of the mortality risk mediated by Glasgow Coma Score (GCS) for each sodium grouping, and show that hypernatremia groups but not hyponatremia groups have a significant percentage of the association between sodium group and mortality mediated by GCS. Sodium category units are in mEq/L

Both hyponatremia (odds ratio [95%CI] for bands: <120mEq/L: 1.58 [1.26–1.97]; 120-<125mEq/L: 1.92 [1.64–2.25]; 125-<130mEq/L: 1.76 [1.60–1.93]; 130–<135mEq/L: 1.32 [1.24–1.41]) and hypernatremia (odds ratio [95%CI] for bands: 140–<145mEq/L: 1.12 [1.05–1.19]; 145-<150mEq/L: 1.89 [1.70–2.11]; ≥150mEq/L: 1.86 [1.57–2.19]) were significantly associated with increased adjusted mortality risk when compared with the reference group of individuals in the 135–<140mEq/L group (Figure 3). GCS treated as a continuous variable differed significantly by dysnatremia group (p<0.001, Table 1). The proportion of individuals with a GCS of 15, considered “no impairment”, was significantly lower in the 145–<150mEq/L and ≥150mEq/L groups (p<0.001), Table 1).

Effect mediation analysis showed decreased CGS had no impact on the relationship between hyponatremia groupings and mortality but mediated over 25% of the association between sodium group and mortality for the 140–<145mEq/L and 145–<150mEq/L groups, and over 50% of the association between the ≥150mEq/L group and mortality (Figure 3). Table 2 shows ADE, ACME, and total effect statistics for each sodium grouping.

## Discussion

Both hypo- and hypernatremia at the time of ICU admission were associated with increased mortality in our cohort, consistent with findings from previous studies [7]. The association between hypernatremia and mortality in our cohort was substantially mediated by level of consciousness, whereas hyponatremia’s association with mortality existed independent of decreased consciousness.

There are a number of potential explanations for the mediating influence of GCS in the hypernatremia groups but not the hyponatremia groups. Neurologic functions responsible for consciousness may be more susceptible to states of water deficiency than states of water excess. There appears to exist a more robust cell volume regulatory mechanism in states of water excess, whereby cells actively extrude osmotically active particles to drive compensatory water egress and restore cell size [1]. Prior studies have suggested the brain and neuromuscular activities are particularly susceptible to hypernatremia [23].

Alternatively, our observations may not reflect the effect of dysnatremia on altered levels of consciousness, but rather the effect of altered levels of consciousness on dysnatremia. Physiologic correction of water deficit in the case of hypernatremia is heavily reliant on a conscious behavior: drinking fluids in response to thirst. With decreased consciousness, individuals are unable to engage in this compensatory behavior. In contrast, physiologic correction of water excess is more heavily reliant on the kidneys’ ability to produce dilute urine, and the conscious behavioral input that can help resolve hyponatremia (consuming more solute) plays a much less significant role in the acute correction stage. The net effect is that decreased consciousness predisposes patients to hypernatremia, not hyponatremia. In this study, because serum sodium and GCS values were extracted on the first day of ICU admission, it is less likely that patients developed hypernatremia due to medically-induced or prolonged periods of altered consciousness impairing their ability to drink, but this nevertheless remains an important consideration.

Our analysis has several important limitations. The myriad mechanisms that orchestrate water retention, including osmolar independent activation of vasopressin as might occur in heart failure, cirrhosis, or in the syndrome of inappropriate antidiuretic hormone release, are not readily measurable and instead rely on the clinical assessment of a patient’s sensed volume status. Given that determining the “sensed” fluid volume remains one of the most challenging aspects in patient care [24], confounding due to underlying illness severity is likely despite efforts to account for it in our model. The predictive value of GCS scoring as a marker for neuronal size and function is not well established. The underlying pathophysiology accounting for dysnatremia is not extractable from the eICU database; more granular data sets with measures of urine concentration and urinary sodium handling would be helpful to further evaluate etiology as it is certainly a contributor to mortality risk. Our analysis does not explicitly take into account the impact of sedating medications on level of consciousness, although sedation for mechanical ventilation purposes is captured by the mechanical ventilation variable.

## Conclusion

Both hyponatremia and hypernatremia are associated with increased mortality in this large cohort of ICU patients, but the association with mortality was mediated by decreased level of consciousness only in the hypernatremic patients. Further studies are needed to characterize the mechanisms by which hypernatremia may create neurologic stressors that ultimately lead to death in order to more concretely establish a causal relationship.

## Figures and Tables

**Table 1:** Study population characteristics stratified by serum sodium category, in 5mEq/L intervals. SD, standard deviation; ICU, intensive care unit; GCS, Glasgow Coma Score; ACS, Acute Coronary Syndrome; CHF, Congestive Heart Failure; CVA, Cerebrovascular accident; GI, gastrointestinal; APACHE 3, acute physiological assessment and chronic health evaluation; IQR, interquartile range.

Variable	Serum Sodium Category (mEq/L)									P
	<120 n = 2447	120–<125 n = 3526	125–<130 n = 11540	130–<135 n = 39890	135–<140 n = 86995 (Ref)	140–<145 n = 43151	145–<150 n = 5952	≥150 n = 2067	Overall n = 195568	
Age in years, mean (SD)	62.1 (16.0)	59.6 (17.5)	61.4 (17.0)	62.7 (17.1)	62.9 (17.4)	63.7 (17.8)	65.2 (17.6)	69.8 (16.3)	63.0 (17.4)	<0.001
Female, n (%)	1249 (51.0%)	1659 (47.1%)	5471 (47.4%)	18113 (45.4%)	39093 (45.0%)	20394 (47.3%)	2771 (46.6%)	921 (44.6%)	89671 (45.9%)	<0.001
Non-white race, n (%)	1975 (80.7%)	2724 (77.3%)	8889 (77.0%)	30728 (77.0%)	67008 (77.0%)	33096 (76.7%)	4417 (74.2%)	1440 (69.7%)	150277 (76.8%)	<0.001
Medical history, n (%)										
Cirrhosis, n (%)	132 (5.4%)	183 (5.2%)	531 (4.6%)	1074 (2.7%)	1369 (1.6%)	741 (1.7%)	158 (2.7%)	28 (1.4%)	4216 (2.2%)	<0.001
CHF, n (%)	305 (12.5%)	496 (14.1%)	2032 (17.6%)	6456 (16.2%)	12561 (14.4%)	6234 (14.4%)	905 (15.2%)	329 (15.9%)	29318 (15.0%)	<0.001
APACHE 3 score, mean (SD)	58.2 (23.4)	63.8 (27.1)	62.2 (27.0)	59.3 (26.5)	55.0 (25.7)	57.7 (27.1)	71.8 (31.0)	86.3 (29.9)	57.9 (26.9)	<0.001
Missing APACHE 3 score, n (%)	163 (6.7%)	284 (8.1%)	858 (7.4%)	3102 (7.8%)	6873 (7.9%)	3419 (7.9%)	514 (8.6%)	195 (9.4%)	15408 (7.9%)	
GCS score, mean (SD)	13.5 (2.79)	13.4 (3.06)	13.4 (3.12)	13.2 (3.25)	13.0 (3.52)	12.4 (3.83)	10.9 (4.14)	9.73 (3.82)	12.8 (3.57)	<0.001
GCS score of 15, n (%)	1491 (60.9%)	2254 (63.9%)	7556 (65.5%)	25069 (62.8%)	52592 (60.5%)	22414 (51.9%)	1719 (28.9%)	243 (11.8%)	113338 (58.0%)	<0.001
Deceased, n (%)	198 (8.1%)	424 (12.0%)	1469 (12.7%)	4089 (10.3%)	6731 (7.7%)	3987 (9.2%)	1122 (18.9%)	449 (21.7%)	18469 (9.4%)	<0.001
Mechanically ventilated, n (%)	332 (13.6%)	596 (16.9%)	2278 (19.7%)	8648 (21.7%)	21501 (24.7%)	13091 (30.3%)	2494 (41.9%)	861 (41.7%)	49801 (25.5%)	<0.001
Serum creatinine in mg/dL, median (IQR)	0.87 (0.58, 1.80)	1.11 (0.71, 2.18)	1.02 (0.75, 2.16)	1.00 (0.77, 1.69)	1.00 (0.75, 1.30)	1.00 (0.75, 1.30)	1.00 (0.78, 1.69)	1.41 (1.00, 2.35)	1.00 (0.75, 1.43)	<0.001
Serum creatinine ≥ 2.0mg/dL, n (%)	505 (20.6%)	913 (25.9%)	3007 (26.1%)	7770 (19.5%)	10397 (12.0%)	4741 (11.0%)	1032 (17.3%)	611 (29.6%)	28976 (14.8%)	<0.001
Admission Diagnosis, n (%)										<0.001
ACS	22 (0.9%)	64 (1.8%)	336 (2.9%)	2133 (5.3%)	5755 (6.6%)	2174 (5.0%)	95 (1.6%)	15 (0.7%)	10594 (5.4%)	
Cardiac arrest	69 (2.8%)	156 (4.4%)	631 (5.5%)	2568 (6.4%)	5973 (6.9%)	3095 (7.2%)	458 (7.7%)	130 (6.3%)	13080 (6.7%)	
CHF exacerbation	93 (3.8%)	149 (4.2%)	594 (5.1%)	1846 (4.6%)	3336 (3.8%)	1736 (4.0%)	246 (4.1%)	29 (1.4%)	8029 (4.1%)	
CVA	37 (1.5%)	98 (2.8%)	367 (3.2%)	2269 (5.7%)	7487 (8.6%)	3553 (8.2%)	281 (4.7%)	57 (2.8%)	14149 (7.2%)	
GI bleed	53 (2.2%)	131 (3.7%)	578 (5.0%)	2168 (5.4%)	4628 (5.3%)	2550 (5.9%)	394 (6.6%)	78 (3.8%)	10580 (5.4%)	
Pneumonia	77 (3.1%)	117 (3.3%)	403 (3.5%)	1505 (3.8%)	2867 (3.3%)	1374 (3.2%)	321 (5.4%)	114 (5.5%)	6778 (3.5%)	
Sepsis	264 (10.8%)	726 (20.6%)	2651 (23.0%)	7325 (18.4%)	10182 (11.7%)	4406 (10.2%)	1127 (18.9%)	704 (34.1%)	27385 (14.0%)	
Trauma	53 (2.2%)	50 (1.4%)	225 (1.9%)	1131 (2.8%)	4507 (5.2%)	2623 (6.1%)	216 (3.6%)	30 (1.5%)	8835 (4.5%)	
Other respiratory disorder	75 (3.1%)	166 (4.7%)	680 (5.9%)	2527 (6.3%)	5573 (6.4%)	2780 (6.4%)	471 (7.9%)	195 (9.4%)	12467 (6.4%)	
Other	1704 (69.6%)	1869 (53.0%)	5075 (44.0%)	16418 (41.2%)	36687 (42.2%)	18860 (43.7%)	2343 (39.4%)	715 (34.6%)	83671 (42.8%)	
ICU type, n (%)										<0.001
Medical	247 (10.1%)	346 (9.8%)	1267 (11.0%)	3808 (9.5%)	6950 (8.0%)	3115 (7.2%)	555 (9.3%)	265 (12.8%)	16553 (8.5%)	
Surgical or med/surg	1658 (67.8%)	2402 (68.1%)	7131 (61.8%)	22915 (57.4%)	48256 (55.5%)	24619 (57.1%)	3630 (61.0%)	1321 (63.9%)	111932 (57.2%)	
Other	542 (22.1%)	778 (22.1%)	3142 (27.2%)	13167 (33.0%)	31789 (36.5%)	15417 (35.7%)	1767 (29.7%)	481 (23.3%)	67083 (34.3%)	

**Table 2:** Causal mediation analysis of the role of altered consciousness in the association between dysnatremia and mortality. Estimates reported are beta values from logistic regression models for association between serum sodium group and mortality. Average Direct Effect (ADE) is the direct effect of sodium group independent of GCS; Average Causal Mediation Effect (ACME) is the indirect effect of sodium group on mortality which is entirely dependent on mediation by GCS; and Total Effect is the sum of direct and indirect effects.

Sodium Band	ADE estimate (95% CI)	ACME estimate (95% CI)	Total Effect estimate (95% CI)
**<120 mEq/L**	0.03 (0.01 to 0.01)	0.00 (0.00 to 0.00)	0.02 (0.01 to 0.04)
**120–<125 mEq/L**	0.03 (0.02 to 0.05)	0.00 (0.00 to 0.00)	0.03 (0.02 to 0.05)
**125–<130 mEq/L**	0.03 (0.03 to 0.04)	0.00 (0.00 to 0.00)	0.03 (0.02 to 0.04)
**130–<135 mEq/L**	0.01 (0.01 to 0.03)	0.00 (0.00 to 0.00)	0.01 (0.01 to 0.02)
**135–<140 mEq/L**	Ref	Ref	Ref
**140–<145 mEq/L**	0.00 (0.00 to 0.01)	0.00 (0.00 to 0.00)	0.01 (0.00 to 0.01)
**145–<150 mEq/L**	0.04 (0.03 to 0.05)	0.01 (0.01 to 0.01)	0.04 (0.04 to 0.05)
**≥150 mEq/L**	0.02 (0.00 to 0.03)	0.02 (0.02 to 0.02)	0.04 (0.03 to 0.06)

## Data Availability

We provide open access to all our data-extraction, filtering, data-wrangling, modeling, figures and tables code and queries at https://github.com/theonesp/sodium_gcs_mortality.

## Take-Home Message

In a population of 195,568 critically ill adults, we found that both hypernatremia and hyponatremia were associated with increased hospital mortality after controlling for multiple demographic and illness severity covariables. Using causal mediation analysis, we show that this association with mortality was significantly impacted by decreased level of consciousness for individuals with hypernatremia, but not hyponatremia, suggesting neurologic dysfunction arising from dysnatremia may be an important driver of mortality in hypernatremic critically ill adults.

## Tweet

↑ and ↓ serum Na → ↑ mortality in critically ill adults. ↓ consciousness mediates this association only in the ↑ Na patients. #bigdata #eICU
